# Editorial: The fertilization success from the Oocyte’s perspective, volume II

**DOI:** 10.3389/fcell.2025.1728990

**Published:** 2025-11-10

**Authors:** Marcela A. Michaut, Joanna M. G. Souza-Fabjan, Rafael A. Fissore

**Affiliations:** 1 Instituto de Histología y Embriología (IHEM), Universidad Nacional de Cuyo, CONICET; Facultad de Ciencias Exactas y Naturales, Universidad Nacional de Cuyo, Mendoza, Argentina; 2 Faculdade de Veterinária, Universidade Federal Fluminense, Niterói, Brazil; 3 Department of Veterinary and Animal Sciences, University of Massachusetts, Amherst, MA, United States

**Keywords:** calcium oscillations, cortical granules, antioxidants, mitochondrial metabolism, vitrification, aquaporin 2

The oocyte remains a central enigma in reproductive biology. Its capacity to integrate multiple layers of signaling, metabolism, and cellular communication defines the success of fertilization and early embryonic development. Following the first volume of this Research Topic, which explored fundamental aspects of oocyte maturation, egg activation, and interaction with the surrounding somatic environment, this second volume expands on our understanding of the oocyte’s journey toward developmental competence. The contributions assembled here reflect both conceptual depth and technological refinement, offering new insights into how molecular, environmental, and physiological cues converge to ensure the initiation of life.

A recurrent theme that unites these studies is the intricate dialogue between the oocyte and its environment—a conversation mediated by ions, organelles, hormones, and signaling molecules. Machaty elegantly revisits one of the most emblematic aspects of fertilization: the calcium signal that awakens the quiescent oocyte. His comprehensive analysis of the mechanisms underlying calcium oscillations not only revisits classical paradigms but also integrates emerging data on sperm-derived factors and intracellular pathways that sustain embryo development. By situating these mechanisms within a comparative framework, this work highlights how nature repurposes and adapts molecular strategies to ensure activation across mammalian species. Complementing this molecular perspective, García-Castro et al. examine the phenogenetics of cortical granule dynamics during the zebrafish’s oocyte-to-embryo transition. Their study bridges genetic and cell biological approaches to elucidate how maternal factors orchestrate cortical exocytosis, a process that safeguards monospermy while remodeling the egg’s surface for the next stage of development. Together, these two contributions underscore that the oocyte is not a passive recipient of fertilizing stimuli, but rather an active participant in its own developmental fate.

Chen et al. present a comprehensive review of the influence of dietary and antioxidant supplementation on oocyte quality. Their work synthesizes evidence showing that compounds such as coenzyme Q10, α-ketoglutarate, melatonin, and omega-3 fatty acids modulate mitochondrial metabolism, oxidative stress, and meiotic competence. The review highlights how nutritional and metabolic interventions can shape the oocyte’s redox balance and developmental potential, emphasizing the emerging role of diet and systemic physiology as critical contributors to reproductive success. By bridging molecular insights and clinical perspectives, this study opens new avenues for improving oocyte competence through targeted nutritional strategies.

Sciorio et al. examine advances in cryopreservation technology, with a focus on human blastocyst vitrification. Their updated review confirms vitrification as the current gold standard, offering superior survival and pregnancy outcomes compared to slow-freezing methods. The authors also discuss the potential biological implications of cryoprotectant exposure, osmotic stress, and temperature fluctuations—particularly regarding oxidative and epigenetic stability. By addressing both technical optimization and safety concerns, this review underscores the need for continued refinement of vitrification protocols to safeguard embryo integrity and long-term developmental health.

Finally, Zheng et al. provide original evidence that connects hormonal signaling to follicular physiology through the regulation of aquaporin 2 (AQP2). By demonstrating that luteinizing hormone modulates AQP2 expression in human granulosa cells via the ERK1/2 pathway, they reveal a new layer of complexity in the control of follicular fluid formation and follicle growth. This discovery not only advances our understanding of the follicular microenvironment but also identifies potential targets for therapeutic intervention in ovarian dysfunction.

Viewed together, these contributions portray the oocyte as a dynamic integrator of signals that extend from the molecular to the systemic level. They remind us that fertilization success depends not solely on the union of gametes but on the precise coordination of events that prepare the egg for that encounter and subsequent development. The studies presented here reflect a field that continues to evolve—where molecular genetics, physiology, and environmental science converge to explain how life begins and sometimes falters.

It becomes evident that the “oocyte’s perspective” is not just a metaphor but a necessary shift in focus. By centering on the female gamete, we gain a holistic view of reproduction—one that acknowledges the vulnerability and resilience of the oocyte within its biological and ecological context. The works in this second volume collectively deepen our appreciation of the complexity of fertilization and open new paths for improving fertility preservation, assisted reproduction, and female reproductive health.

